# A Bill to Establish a Department of Public Health

**Published:** 1894-03

**Authors:** 


### A BILL TO ESTABLISH A DEPARTMENT OF PUBLIC HEALTH.

*Be it enacted by the Senate and House of Representatives of the United States in Congress assembled:*

SECTION 1. That there shall be established a Department of Public Health. There shall be appointed by the President from the medical profession, by and with the advice and consent of

---

the Senate, a Secretary of Public Health, who shall be entrusted with the management of the department herein established. He shall be paid an annual salary of $8000.

There shall be appointed by the President, with the approval of the Senate, an Assistant Secretary of Public Health, at an annual salary of $5000.

The Secretary of Public Health shall, with the approval of the President, provide suitable offices for the department, and shall employ such assistants and clerks as may be necessary.

SEC. 2. It shall be the duty of the Secretary of Public Health to obtain through all accessible sources, including State boards of health, municipal authorities, and the Surgeon-Generals of the Army, Navy and Marine Hospital Service of the United States, weekly reports of the sanitary condition of all ports and places within their territories and departments, and he shall publish weekly abstracts of the information thus obtained and other pertinent matter received by his department. The said department also shall, as far as possible, by means of the voluntary co-operation of State and municipal authorities, of various general and special hospitals, sanitariums, public associations and private persons, procure and tabulate statistics of marriage, births (noting those that are illegitimate), and deaths from epidemic, endemic and all other diseases, specifying those of a degenerative character, such as malignant growths, and affections of the nervous, circulatory, respiratory, secretory, digestive and reproductive organs, and from violence, accidents, suicide, murder, and data concerning the fruit of consanguineous marriages, and the transmissibility of insane, alcoholic, syphilitic, nervous and malignant types of constitution to offspring, and to evils of race miscegenation. He shall also procure information relating to climatic and other conditions beneficial to health, and especially in reference to the most favorable regions in the United States for the cure or relief of chronic diseases, particularly tubercular consumption. He shall also procure information as to the prevalence and ruinous effects upon the body and mind of intemperance and prostitution. He shall endeavor to ascertain the extent, the origin and classification of insanity in the several States and Territories of the country. He shall investigate the state of comfort of the laboring classes in respect to their lodgment, their trades, occupations, the healthfulness of their workshops and the contents of the atmosphere they habitually breathe, and the prevalence of premature degeneration of the nervous and muscular systems by the exactions of piece-work employment. He shall obtain information in regard to the soundness of their food and purity of water-supply. He shall ascertain the ages at which the children of the poor are put to work, and its hindrance to their physical development, and their lack of common-school education. He shall seek through the State boards of health information of the hygienic state of public school buildings respecting their illumination, ventilation, and the presence of noxious elements in the circumambient air. He shall seek information in regard to the pollution of streams and navigable waters and public and private wells. He shall attempt, through the co-operation of the authorized medical schools in all the States, to promote the most extended and thorough training of students in order to fit them for the reponsible duties that devolve upon practitioners of medicine. He shall, whenever an epidemic disease is spreading abroad, or in any country which by commercial or other relations may endanger the health of the inhabitants of the United States, have power to call a conference of the Surgeon-Generals of the Army, Navy and Marine Hospital Service, and the executive officer or officers of the various State boards of health throughout the country, to consider and advise with him in regard to the best methods to be pursued to protect the country against the invasion of any such epidemic disease, and the results of such conference shall be, by the Secretary of Public Health, communicated to the President and his Cabinet, for such action as they may deem wise and expedient.

Besides the reports of the state of the public health which he shall make from time to time, the Secretary shall make an annual report to Congress, with such recommendations as he may deem important to the public welfare, and the report, if ordered printed by Congress, shall be done under the direction of the department. The necessary printing of the department shall be done at the government printing office upon the requisition of the Secretary, in the same manner and subject to the same provisions as that of other printing for the several departments of the government.

SDC. 3. The President is authorized, when requested by the Secretary of Public Health, and when the same can be done without prejudice to the public service, to detail officers from the several departments of the government for temporary duty, to act under the Department of Public Health to carry out the provisions of this act, and such officers shall receive no additional compensation except for actual and necessary expenses incurred in the performance of such duties. When a detail of such officers cannot be made, the Secretary, approved by the President, may employ such experts, and for such time and in such manner as the funds at the disposal of the department may warrant.

SEC. 4. That to defray the expenses in carrying out the provisions of this act, the sum of fifty thousand dollars ($50,000), or as much thereof as may be neccssary, is hereby appropriated to be disbursed with the approval of the President, under the Secretary of said department.

This act shall take effect sixty days after its passage, within which time the Secretary and Assistant Secretary may be appointed.*

*This is the American Medical Association's bill, now before Congress.

